# Gender and birth weight as risk factors for anastomotic stricture after esophageal atresia repair: a systematic review and meta-analysis

**DOI:** 10.1186/s12887-020-02295-3

**Published:** 2020-08-24

**Authors:** Anahid Teimourian, Felipe Donoso, Pernilla Stenström, Helena Arnadottir, Einar Arnbjörnsson, Helene Lilja, Martin Salö

**Affiliations:** 1 Department of Clinical Sciences, Pediatrics, Lund University, Lund, Sweden; 2 Department of Women’s and Children’s Health, Pediatric Surgery, Uppsala University, Uppsala, Sweden; 3 Department of Pediatric Surgery, Uppsala University Hospital, Uppsala, Sweden; 4 Department of Pediatric Surgery, Skåne University Hospital, Lasarettsgatan 48, 221 85 Lund, Sweden

**Keywords:** Anastomotic stricture, Birth weight, Esophageal atresia repair, Gender, Meta-analysis, Risk factors

## Abstract

**Background:**

Anastomotic stricture (AS) is the most frequently occurring complication that occurs after esophageal atresia (EA) repair. Nevertheless, the pathogenesis remains primarily unknown and there is inadequate knowledge regarding the risk factors for AS. Therefore, a systematic review of the literature and a meta-analysis was performed to investigate whether gender and birth weight were risk factors for the development of AS following EA repair.

**Methods:**

The main outcome measure was the occurrence of AS. Forest plots with odds ratios (OR) and 95% confidence intervals (CI) were generated for the outcomes. Quality assessment was performed using the Newcastle–Ottawa scale.

**Results:**

Six studies with a total of 495 patients were included; 59% males, and 37 and 63% of the patients weighed < 2500 g and ≥ 2500 g, respectively. Male gender (OR, 0.96; 95% CI, 0.66–1.40; *p* = 0.82) and birth weight < 2500 g (OR, 0.74; 95% CI, 0.47–1.15; *p* = 0.18) did not increase the risk of AS. The majority of the included studies were retrospective cohort studies and the overall risk of bias was considered to be low to moderate.

**Conclusion:**

Neither gender nor birth weight appear to have an impact on the risk of AS development following EA repair.

## Background

Esophageal atresia (EA) is a rare congenital anomaly that occurs in 1:2500 to 1:4000 live-born children [1]. The survival rate has increased to up to 91–99% in the past decade [2–5]. Excluding the immediate postoperative complications, the most frequently occurring complication affecting postoperative morbidity is the development of anastomotic strictures (AS) [6]. The rate of AS after EA repair varies with different studies, and a universal definition is lacking; however, approximately 32–59% of children are expected to require at least one dilatation during their lifetime [6].

Only a few risk factors for developing AS are known thus far, and their incidence may be affected by the type of EA. Long-gap EA, which is exposed to increased tension in the anastomosis, is considered to be more likely to form AS; in addition, recent studies confirm that anastomotic tension is an independent risk factor of AS development [7, 8]. Earlier research also suggested that AS might be influenced by gastro-esophageal reflux (GER), regardless of the presence of anastomotic tension [5]. However, recent studies have indicated that anti-reflux medication does not reduce the development of AS; nonetheless, proton pump inhibitors (PPI) are still used, possibly because they are considered to be harmless [8, 9].

Several studies have been conducted [6] regarding the incidence of AS after EA repair, and many of them have included gender and birth weight while describing the characteristics of the patients. However, the correlations between these two parameters and AS formation have not been evaluated so far. There are studies indicating that gender may play a role for length of stay after repair of esophageal atresia [10], and for overall morbidity and outcome after surgery in children [11, 12]. In the present study, we aimed to systematically review and perform a meta-analysis of the literature to analyze whether gender and birth weight are risk factors for the development of AS.

## Methods

### Search strategy

The PRISMA (Preferred Reporting Items for Systematic Reviews and Meta-Analyses) guidelines were followed [13]. The literature was searched using Embase, PubMed, and Cochrane. The search terms ‘esophageal atresia’ and ‘esophageal atresia’ were used. These terms were then combined with ‘anastomotic stricture’, ‘anastomotic stenosis’, ‘sex’, ‘gender’, and ‘birth weight’ in order to narrow down the search. The search function included ‘all fields’. Filters were set for articles published during the years 2000–2019 and the language was set to English and French. Only original articles were selected. Articles published earlier than 2000 and in other languages were excluded (Table 1). The initial selection was performed by screening the title and/or the abstract for studies involving AS after EA repair. AS was not defined prior to the search due to the lack of a uniform definition*.* The articles matching the inclusion criteria were then retrieved in full text. Secondary selection was performed by screening the patient characteristics in the selected literature. The data required were the number of males/females who developed AS and the birth weights of patients who did and did not develop AS. The articles had to include at least one of the two criteria in order to be included in this study.

**Table 1:** Search strategy, terms, inclusion criteria, and search results in the present study

Search no.	Terms used	Inclusion criteria	Search results				Sample 1	Sample 2
			Embase	PubMed	Cochrane	Total	50	6
1	(Esophageal atresia OR esophageal atresia) AND anastomotic stricture	Published 2000–2019, English/French	131	149	33	313		
2	(Esophageal atresia OR esophageal atresia) AND anastomotic stenosis	Published 2000–2019, English/French	32	109	32	173		
3	(Esophageal atresia OR esophageal atresia) AND (anastomotic stricture OR anastomotic stenosis) AND (birth weight) AND gender	Published 2000–2019, English/French	1	4	NA	5		
4	(Esophageal atresia OR esophageal atresia) AND (anastomotic stricture OR anastomotic stenosis) AND (birth weight) AND sex	Published 2000–2019, English/French	5	1	37	43		
5	(Esophageal atresia OR esophageal atresia) AND (anastomotic stricture OR anastomotic stenosis) AND (birth weight)	Published 2000–2019, English/French	28	24	NA	52		
6	(Esophageal atresia OR esophageal atresia) AND (anastomotic stricture OR anastomotic stenosis) AND (gender OR sex)	Published 2000–2019, English/French	11	7	NA	18		
Total			208	294	104	604		

*NA* Not available

The initial screening and first selection were performed by two authors (AT and MS). The secondary selection was performed by the same two authors and supervised by a third (EA). Disagreements were resolved with discussion between the three authors (AT, MS, EA).

### Data extraction

The data extracted for analysis were author, year of publication, study period, gender, birth weight, and rate of stricture formation after EA repair. Centers from two of the studies [8, 11] (Table 2) supplied additional data, making it possible for inclusion in the analysis.

**Table 2:** Summary of included articles evaluating the impact of sex and birth weight on the risk of developing anastomotic strictures after repair of esophageal atresia

Study (year)	Study design	Country	Study period	Sample size	Reported stricture rate (%)		
					M/F	BW < 2500 g	BW > 2500 g
Michaud et al. (2001) [14]	Retrospective cohort single-center	France	(Five years)	50	37/45	30	47
Allin et al. (2014) [15]	Prospective cohort multi-center	UK and Ireland	2008–2009	76	40/34	28	43
Nice et al. (2016) [16]	Retrospective cohort single-center	USA	1999–2014	121	20/24	NA	NA
Okata et al. (2016) [7]	Retrospective cohort single-center	Japan	2000–2015	28	53/31	NA	NA
Stenström et al. (2017) [17]	Retrospective Case-Control single-center	Sweden	Case 2010–2014, Control 2001–2009	93	39/42	43	42
Donoso et al. (2017) [8]	Retrospective cohort single-center	Sweden	Case 2005–2013, Control 1994–2004	126	53/56	51	56

*M* Male; *F* Female, *BW* Birth weight; *NA* Not available

### Quality assessment

For assessment of the risk of bias in the included studies, the Newcastle-Ottawa scale (total of nine stars), which evaluates three major aspects of quality including selection, comparability, and outcome for cohort studies [18], was used. Studies with a low risk of bias were allocated ≥7 stars, moderate risk with 4 to 6 stars, and high risk with ≤3 stars.

### Statistics

The statistical analysis was performed using Review Manager 5.3 (Copenhagen, Denmark).

All data were analyzed using dichotomous variables. One of the articles^13^ had to be transformed from continuous to dichotomous variables. This was performed manually by using the normal distribution graph. The cut-off for birth weight was 2500 g. AS development was the main outcome measure in the study. The exposures were male gender and low birth weight (< 2500 g). Forest plots were generated in which, the pooled odds ratios (OR) were calculated for each article by using the Mantel–Haenszel method [19], with a confidence interval (CI) of 95%. Ultimately, the summary effect measure (OR) was calculated with a 95% CI. The significance level was set to *p* < 0.05. No funnel plots were created due to the low number (< 10) of included articles.

## Results

**Fig. 1:**
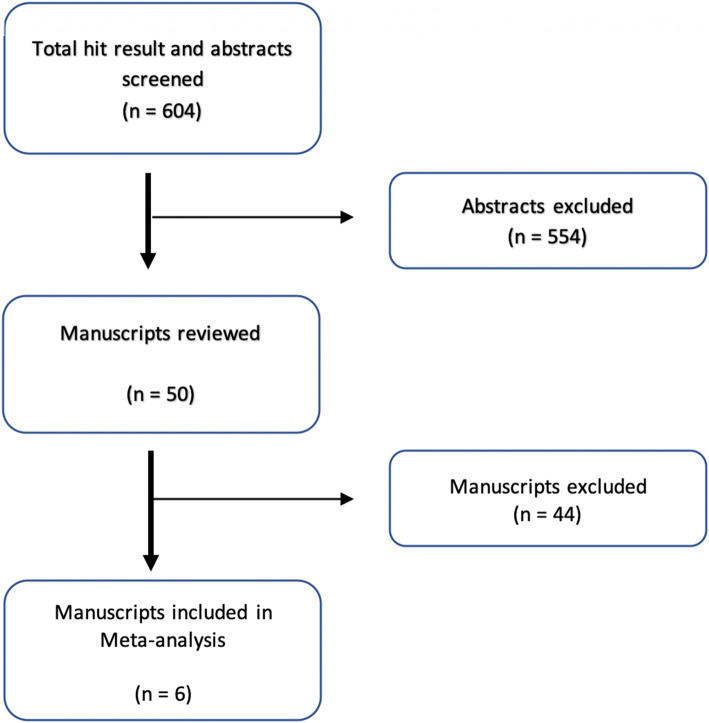
Flowchart of the search process for articles evaluating the effect of gender and birth weight on the risk of developing anastomotic strictures after the repair of esophageal atresia

A total of 604 articles were found in the Embase, PubMed, and Cochrane database (Table 1). Fifty articles were obtained initially, after narrowing down the number of articles using the selected search terms (Fig. 1). After reviewing the patient characteristics in the chosen articles, eight articles were found to qualify for the meta-analysis; two of them belonged to the same cohort [10, 17]. Finally, the study by Stenström et al. [17] was chosen because it included more patients. Full texts of the remaining seven articles were read, and, ultimately, six articles [7, 8, 14–17] were included in the meta-analysis (Table 1; Fig. 1).

Only one article was focused solely on EA type C [17]. The remaining five articles were also predominantly focused on type C cases (ranging between 82 and 94%), but included other Gross types as well [7, 8, 14–16]. The age at first dilation was reported in five articles with a median age of 2 to 6 months. The number of dilations needed was presented in different time periods, mainly until 1 year of age; however, Donoso et al. reported a 5-year follow-up. In four [8, 15–17] of the studies, the median number of dilations needed ranged between two to three. Okata et al. [7] did not report this parameter, whereas Michaud et al. [14] reported a median of seven dilatations.

**Fig. 2:**
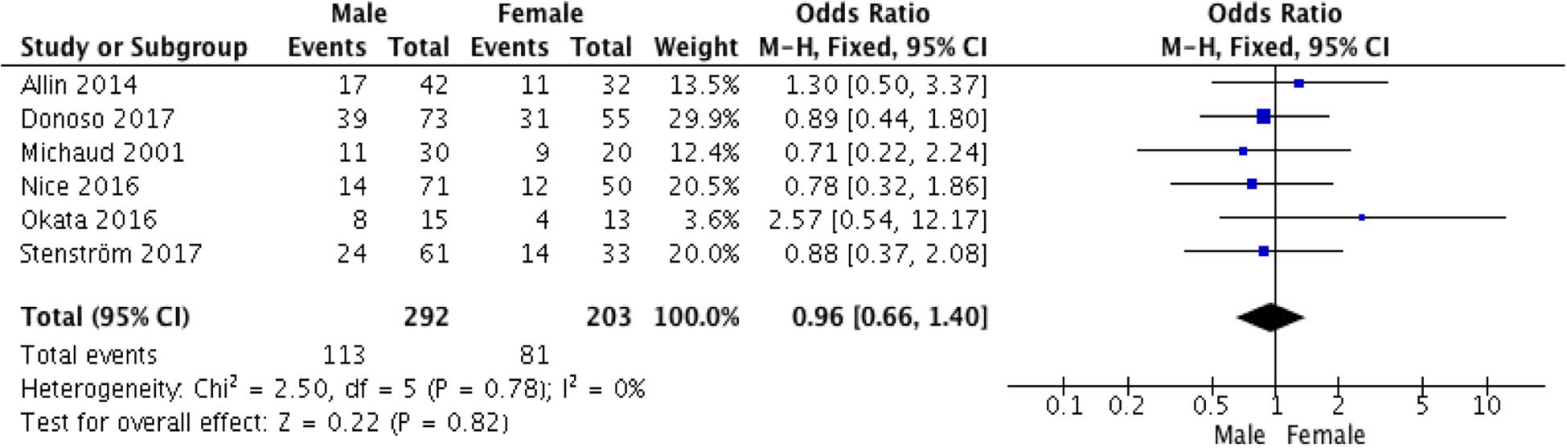
Forest plot of the impact of male gender on the risk of developing an anastomotic stricture after repair of esophageal atresia. CI: confidence interval; M-H: Mantel–Haenszel method

All six articles [7, 8, 14–17] analyzed the impact of gender on AS. A total of 495 (range 28 to 126 per article) patients (males, 292; females, 203) with EA were included; among them, 113 (39%) and 81 (40%) males and females, respectively, developed AS (Table 2). The summary effect measure was as follows: OR, 0.98; 95% CI, 0.67–1.43 (Fig. 2).

**Fig. 3:**
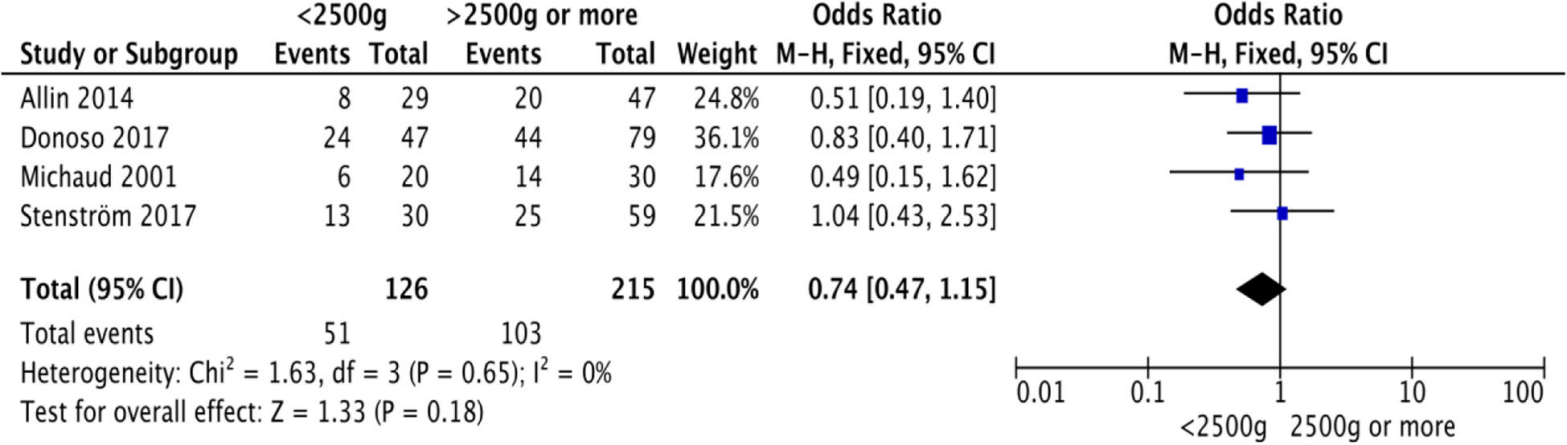
Forest plot of the impact of low birth weight (< 2500 g) on the risk of developing an anastomotic stricture after repair of esophageal atresia. CI: confidence interval; M-h: Mantel–Haenszel method

Four articles [8, 14, 15, 17] could be used to analyze the impact of birth weight on AS development. Thus, the total number of patients evaluated was 341, 126 (37%) of who had a birth weight of < 2500 g, while the remaining 215 (63%) had a birth weight of ≥2500 g. A total of 51 (40%) and 56 (48%) patients in the < 2500 and > 2500 g birth weight groups, respectively, developed AS (Table 2). The summary effect measure was: OR, 0.74; 95% CI, 0.47–1.15 (Fig. 3).

Although all studies (except for one [15]) were retrospective cohort studies, the overall risk of bias was considered to be low to moderate using the Newcastle-Ottawa scale for the assessment of the risk of bias. Four studies scored ≥7 stars indicating a low risk of bias and two scored 6 stars indicating a moderate risk of bias (Table 3).

**Table 3:** Assessment of the included studies using the Newcastle-Ottawa scale

Study	Selection				Comparability	Outcome				Score
	Representativeness of the exposed cohort	Selection of the non-exposed cohort	Ascertainment of exposure	Demonstration that the outcome of interest was not present at the start of the study	Comparability of cohorts on the basis of the design or analysis	Outcome	Assessment of outcome	Follow-up long enough for outcomes to occur	Adequacy of follow-up of cohorts	
Michaud et al. (2001) [14]	+	+	+	+	–	Stricture	+	+	–	6
Allin et al. (2014) [15]	+	+	+	+	–	Stricture	+	+	+	7
Nice et al. (2016) [16]	+	+	+	+	–	Stricture	+	+	–	6
Okata et al. (2016) [7]	+	+	+	+	+	Stricture	+	+	+	8
Stenström et al. (2017) [17]	+	+	+	+	+	Stricture	+	+	+	8
Donoso et al. (2017) [8]	+	+	+	+	++	Stricture	+	+	–	8

## Discussion

In this meta-analysis, neither gender nor birth weight was found to have an impact on the risk of developing AS after EA repair.

In total, six articles [7, 8, 14–17] were found to match our criteria for analysis of the impact of gender on AS development. The pooled OR was approximately 1, which suggests no significant difference between males and females with regard to the risk of developing AS. The rate of AS was higher in females in four [8, 14, 16, 17] out of six articles, ranging between 3 to 8 percentage points compared to males. On combining the data from all six studies, [7, 8, 14–17] an overall rate of 40% in females and 39% in males was observed.

Furthermore, the pooled OR for AS, based on birth weight, did not differ significantly. Only four articles [8, 14, 15, 17] were suitable for birth weight analysis. Difficulties in analyzing this parameter were encountered due to diverse cut-offs on birth weight in the different articles. In one article [14], manual conversion from nominal to categorical data was performed based on the normal distribution. This may have compromised the accuracy of the original data. In three [8, 15, 16] out of the four articles, the rate of AS was higher (range, 5–17%) in patients with ≥2500 g birth weight compared to those with < 2500 g birth weight.

Thus, neither birth weight nor gender seemed to impact the development of AS. It is worth noting that the included studies were mainly retrospective and single-cohort studies. However, the risk of bias was considered to be low to moderate when assessed with the Newcastle-Ottawa scale. There was also the risk of type II error due to the low number of articles included. Follow-up time is an important parameter that needs to be considered while evaluating the rate of AS. Two of the selected articles [14, 16] failed to report the follow-up time. The remaining four [7, 8, 15, 17] had a follow-up period of 1 year after the EA repair. Of the six articles, only one [7] did not present the age at first dilatation or the number of dilations required in the cohort. The wide variations in these two parameters in the remaining articles might indicate the differences in the criteria for requiring dilation and the definition of AS. As noted in the introduction, the definition of AS is not universal. The articles chosen in the current study presented different definitions, which may confound the results. In addition, it might explain the wide range in AS rate in the included studies. Most studies primarily relied on symptoms that were confirmed with an esophagram. Michaud et al. [14] included failure to thrive in their definition of AS. Thus, a universal definition of AS will be of great value in the future.

AS is one of the main causes of morbidity after EA reconstruction. Therefore, it is important to explore the risk factors for the underlying mechanism. The ability to predict the development of AS after EA repair might prove useful for a safe postoperative follow-up and high-quality parental information. Identification of risk factors and an improved understanding of the pathogenesis of AS could aid in the development of preventative therapies.

The main strength of this study is that it explored a field that has not been researched in detail or reviewed systematically, so far. Most of the articles included in this meta-analysis were recently published, except for one [14]. The latter provides a current depiction of this particular research field and its upcoming challenges. Another strength of our study was that all the articles selected were from different countries. This gives a worldwide perspective of the EA and AS rates based on the gender and birth weights of the patients. It also manifests the differences in the definitions of AS, which is a topic of discussion in several published studies [6].

Our study had various limitations. First, the number of patients in all the selected articles was low. EA is an uncommon congenital malformation and thus, understandably, most studies are small cohort studies. Second, based on the method used in our study, selection bias may be a confounder. In general, the articles found during the first selection did not tabulate the patients’ characteristics in a detailed manner, based on our requirements. This may have excluded articles that could potentially have had added to the raw data in the current study. We tried to contact the authors in order to receive more elaborate raw data but were only successful with two authors [8, 17]. Third, the data from the selected articles were predominantly based on EA Gross type C but not in full effect. It was also impossible to adjust for age at surgery; hence, possibly later repair due to long gap often have more tension in the anastomosis and consequently have higher risk of anastomotic stricture. Although randomized control trials were not applicable in this meta-analysis, all articles, except one, were single-center retrospective cohort studies. Further research using a different approach, such as generating a multi-centered database on patient characteristics and focusing on specific types of EA, may be useful. A larger database could then lead to less selection bias and a better chance of evaluating the impact of gender and birth weight on the development of AS.

## Conclusion

This meta-analysis studied esophageal AS, the main complication after EA reconstruction. Although males are more likely to develop EA, they do not have a higher risk of AS. Furthermore, birth weight does not seem to be a risk factor for developing AS after reconstruction of EA. Further studies with larger sample sizes are required to analyze these two parameters in detail, which may potentially aid in the early detection of children who are at risk of developing AS.

## Data Availability

All data generated or analysed during this study are included in this published article.

## Abbreviations

AS: Anastomotic stricture
EA: Esophageal atresia
GER: Gastro-esophageal reflux
PPI: Proton pump inhibitors
TEF: Tracheoesophageal fistula
